# Objective motor function assessment using diffusion tensor tractography in subacute acquired brain injury: A retrospective observational study

**DOI:** 10.1097/MD.0000000000046721

**Published:** 2025-12-26

**Authors:** Au-Jin Wang, Choong-Hee Roh, Myoung-Hwan Ko, Da-Sol Kim, Yu Hui Won, Sung-Hee Park, Jeong-Hwan Seo, Gi-Wook Kim

**Affiliations:** a Department of Physical Medicine & Rehabilitation, Jeonbuk National University Medical School, Jeonju, Republic of Korea; b Research Institute of Clinical Medicine of Jeonbuk National University-Biomedical Research Institute of Jeonbuk National University Hospital, Jeonju, Republic of Korea.

**Keywords:** corticospinal tract, Diffusion tensor tractography, fractional anisotropy, motor function, prognosis, subacute brain injury

## Abstract

Corticospinal tract (CST) integrity is a key determinant of motor recovery after focal brain injury. Diffusion tensor tractography (DTT) can quantify CST microstructural integrity, but clinically applicable thresholds predicting tract integrity to motor function remain unclear. This study aimed to evaluate whether DTT-derived CST fiber number (FN) and fractional anisotropy (FA) ratios (affected/unaffected, A/U) can serve as objective indicators of motor function in patients with subacute focal acquired brain injury, and to determine optimal cutoff values for these metrics. We retrospectively analyzed 70 patients (7–90 days post-onset) with first-ever unilateral hemiparesis due to ischemic stroke, intracerebral hemorrhage, subarachnoid hemorrhage, hypoxic brain injury, or brain tumor. Motor function was assessed using the Medical Research Council (MRC) scale, hand function tests (HFTs), and the Modified Barthel Index (MBI). CST FN and FA values were measured on both sides, and ratios (A/U) were calculated. Wilcoxon signed-rank tests compared affected versus unaffected sides. Receiver operating characteristic (ROC) analyses with 95% confidence intervals (CIs) identified optimal CST ratio cutoffs associated with neurophysiologic and functional milestones. The CST FN and FA values were significantly reduced on the affected side (all *P* < .001). Higher FN and FA ratios (A/U) correlated with larger motor-evoked potential amplitudes, higher MRC grades, higher HFT scores, and better MBI outcomes. ROC analysis yielded clinically applicable cutoff values that increased with task difficulty. DTT-derived CST FN and FA ratios (A/U) provide quantitative, objective indicators of motor function in subacute focal brain injury. These tract-based metrics may assist in clinical decision-making and rehabilitation planning by offering reproducible thresholds linked to functional performance.

## 1. Introduction

Acquired brain injury, including ischemic or hemorrhagic stroke, subarachnoid hemorrhage, hypoxic brain injury, and brain tumor, is a common cause of motor impairment, in which patients often experience residual weakness and fine motor deficits.^[1,2]^ Although these etiologies are heterogeneous, they share the potential to involve the corticospinal tract (CST), the principal descending pathway responsible for voluntary movement and fine motor control.^[3]^ Once the CST is affected, patients typically develop varying degrees of motor weakness and consequent functional limitation depending on the extent of tract damage. Among these etiologies, stroke, particularly middle cerebral artery (MCA) infarction, is well known to frequently involve the CST.^[4,5]^ In contrast, mild traumatic brain injury (concussion) generally spares the tract, whereas moderate to severe traumatic brain injury often results in CST disruption secondary to diffuse axonal injury, leading to motor deficits and slowed recovery.^[6]^ In brain tumors, CST injury may occur due to direct invasion, peritumoral edema, or compression effects related to lesion-to-tract distance.^[7]^ In subarachnoid hemorrhage, vasospasm-induced ischemia can lead to secondary CST damage,^[8,9]^ while in hypoxic brain injury, diffuse white matter hypoxia may result in structural injury to the tract.^[10]^

Early rehabilitation is essential to minimize these functional deficits, and the ability to predict motor strength and functional performance during the initial evaluation is an important factor in establishing appropriate rehabilitation goals.^[11]^ The CST, also known as the pyramidal tract, transmits voluntary motor commands from the motor cortex to spinal motor neurons, and its integrity serves as a critical biomarker for predicting motor recovery after brain injury.^[12]^

Previous studies have attempted to predict functional recovery by assessing both the electrophysiological and structural integrity of the CST. Motor-evoked potentials (MEPs), which reflect the excitability of the corticospinal pathway, have been widely used to predict motor outcomes after acquired brain injury.^[13,14]^ Positive MEP responses are generally associated with favorable functional recovery, whereas absent responses indicate poor prognosis.^[13,15]^ Moreover, MEP amplitude can serve as a quantitative indicator of residual motor function in patients with stroke.^[16]^

In parallel, diffusion tensor imaging (DTI)–based tractography has emerged as a complementary tool to evaluate the anatomical status of the CST. Numerous studies have demonstrated correlations between DTT-derived CST metrics and motor recovery, suggesting that these parameters can serve as imaging biomarkers for predicting functional outcomes after acute and subacute acquired brain injury.^[17–20]^ However, only a few studies have proposed objective quantitative indices from DTT parameters that directly represent motor function.

Unlike previous studies that focused on a single etiology, our study included patients with various forms of acquired brain injury to comprehensively assess how the integrity of the CST correlates with gradations of motor functional ability. We further identified the optimal cutoff values of fiber number (FN) and fractional anisotropy (FA) ratios on DTT corresponding to different levels of motor difficulty, such as grip movement, evoked MEP response, and performance on hand and lower-limb functional tasks.

## 2. Materials and methods

### 2.1. Subjects

We retrospectively reviewed the medical records of 70 inpatients with asymmetric motor weakness caused by subacute focal brain injury who underwent DTI, MEP, and functional assessment scales at Jeonbuk National University Hospital between January 2020 and December 2022. All patients had been admitted to the Department of Rehabilitation Medicine for intensive inpatient rehabilitation and had completed transcranial magnetic stimulation (TMS) for motor-evoked potential (MEP) assessment and standardized functional evaluations including hand function tests (HFTs) and the Modified Barthel Index during their hospital stay.

The subacute phase was defined as 7 to 90 days after onset. Patients were included if they were adults (≥18 years old), had asymmetric motor weakness with one side showing reduced motor strength compared to the contralateral side as confirmed by neurological examination, were able to undergo both DTI and TMS evaluations within the subacute period, and had no prior history of central nervous system disease.

Exclusion criteria were as follows: Patients with symmetrical or equally severe bilateral motor weakness, recurrent stroke or previous brain injury, severe cognitive impairment or poor cooperation that precluded reliable motor assessment, contraindications to TMS (e.g., metallic implants, seizure disorder), and incomplete or missing medical records.

This study was approved by the Institutional Review Board of Jeonbuk National University Hospital (CUH 2021-01-040), and all procedures were conducted in accordance with the Declaration of Helsinki.

### 2.2. Diffusion tensor image

DTI data were retrospectively obtained from clinically acquired brain MRI scans that had been performed for diagnostic purposes using a 3.0 T Siemens Verio scanner (Siemens, Erlangen, Germany) equipped with a single-shot echo-planar imaging sequence and 2 diffusion-sensitizing gradients. All DTI scans and tractography reconstructions were originally conducted as part of routine clinical evaluations, independent of this research. For the present study, these previously acquired DTI datasets and tractography results were retrospectively reviewed and reanalyzed.

To reduce scan time and minimize image distortion, the Generalized Autocalibrating Partially Parallel Acquisition technique had been applied during image acquisition, and automated registration software was used for eddy-current and motion correction.

The imaging parameters were as follows: echo time = 105 ms; repetition time = 7000 ms; field of view = 200 × 200 mm²; matrix size = 128 × 128; number of excitations = 2; b = 1000 s/mm²; 30 diffusion directions; slice thickness = 3.0 mm; and 46 contiguous slices parallel to the anterior commissure–posterior commissure (AC–PC) line.

The CST was reconstructed using DTI Studio software (v.1.02, Johns Hopkins Medical Institute, Baltimore, MD, USA) following standard clinical protocols. For CST reconstruction, regions of interest (ROIs) were manually placed on color-coded FA maps: the seed ROI in the CST region at the anterior mid-pons and the target ROI in the precentral gyrus corresponding to the primary motor cortex. Fiber tracking was terminated at a FA threshold of 0.2 and an angular deviation > 60°, and only fibers passing through both ROIs were included.

For quantitative analysis, the FN and FA values of the CST were calculated separately for the affected and unaffected hemispheres, and FN and FA ratios (affected/unaffected) were used to account for inter-individual variability.^[17,21]^

### 2.3. Transcranial magnetic stimulation

TMS data were retrospectively obtained from examinations that had been performed during the inpatient rehabilitation period as part of routine clinical evaluation. TMS was conducted using a Magstim 200 stimulator (Magstim, Whitland, Wales, UK) equipped with a 65-mm figure-eight coil connected to a Medtronic Keypoint® E electromyography unit (Medtronic Inc., Skovlunde, Denmark). The signal had been digitized at 10 kHz, with a high-pass filter of 100 Hz and a low-pass filter of 5 kHz.

Motor-evoked potentials (MEPs) were originally recorded from the first dorsal interosseous (FDI) and tibialis anterior (TA) muscles following stimulation of the contralateral primary motor cortex. Cortical mapping had been performed along a 2 × 2 cm grid over the hand and leg areas at rest, with the coil handle oriented posteriorly at 45° from the midline to induce posterior–anterior current flow. Both hemispheres were examined to compare ipsilateral and contralateral responses.

For each MEP detected, the optimal scalp site that produced consistent muscle responses at the lowest stimulation intensity and the resting motor threshold (RMT) were identified. The RMT was defined as the minimum intensity that elicited at least 5 MEPs > 50 μV in 10 trials.^[22]^ MEP amplitudes were measured from responses evoked at 120% of the individual RMT. Each stimulation site had been tested 4 times, and the shortest latency and mean peak-to-peak amplitude were used for analysis.

The MEP amplitude ratio was calculated as the affected-to-unaffected side ratio to reflect the relative corticospinal excitability of the lesioned hemisphere.^[13]^ All TMS and MEP data were retrospectively reviewed and analyzed in relation to the DTI-derived FN and FA ratios of the CST and the functional outcome measures.

### 2.4. Functional assessment

Functional evaluation data were retrospectively collected from assessments that had been performed during the inpatient rehabilitation period once patients were medically and neurologically stable. These evaluations had been routinely conducted on the day of admission and on the day of discharge, following approximately 3 weeks of rehabilitation, to document post-rehabilitation functional status. For the present study, the data were retrieved from medical records and the hospital’s functional assessment database. All assessments had been conducted by trained rehabilitation physicians and occupational therapists who were blinded to the DTI results, using standardized and validated clinical scales to minimize subjectivity.

The Medical Research Council (MRC) scale was used to grade the muscle strength of finger flexion and extension and ankle dorsiflexion on a scale from 0 (no contraction) to 5 (normal strength).

HFTs quantified upper-limb motor performance, including grip strength, the Nine-Hole Peg Test (NHPT), and the Box and Block Test (B&B).^[23]^ Grip strength was measured using a JAMAR hydraulic hand dynamometer (Sammons Preston, Chicago, IL, USA), recording the best of 3 trials. The B&B test assessed gross manual dexterity by counting the number of blocks transferred between compartments within 1 minute, and the NHPT evaluated fine motor dexterity by timing the placement and removal of 9 pegs, with shorter completion times indicating better function. All tests had been conducted in a standardized seated position, and inter-rater reliability was excellent (intraclass correlation coefficient > 0.9).

Activities of daily living were evaluated using the Korean version of the Modified Barthel Index (K-MBI), which measures 10 items, personal hygiene, bathing, feeding, toileting, dressing, bowel and bladder control, stair climbing, ambulation, and chair-bed transfer.^[24]^ The K-MBI has been validated in Korean patients with stroke and brain injury and reflects the degree of independence in daily activities following subacute rehabilitation.

### 2.5. Statistical analysis

All statistical analyses were performed using SPSS version 23.0 for Windows (SPSS Corp., Armonk). Comparisons between the affected and unaffected sides for MEP amplitude, functional assessments, and DTI parameters (FN and FA of the CST) were performed using the Wilcoxon signed-rank test.

The FN, FA, and MEP amplitude ratios were calculated by dividing the affected-side value by the unaffected-side value. ROC curve analyses were conducted to determine the optimal cutoff scores for the FN and FA ratios of the CST and for the MEP amplitude ratio in relation to motor and functional outcomes. Positive outcomes were defined as the ability to perform the corresponding function, including MRC ≥ 2 for limb strength, measurable grip strength, completion of HFTs, and ambulation or stair climbing with at least moderate assistance according to the K-MBI. Negative outcomes were defined as inability to perform the task, MRC < 2 for limb strength, or requiring maximal assistance. The area under the curve (AUC) and its 95% confidence interval (CI) were calculated to evaluate the discriminative power of each parameter.

Correlation analyses were performed to examine the relationships between CST integrity indices (FN and FA ratios), MEP amplitude ratios, and functional outcome measures, including MRC grades, HFTs (grip strength, Box and Block Test, and Nine-Hole Peg Test), and Modified Barthel Index (MBI) scores. The MBI was further subdivided into upper- and lower-limb subscores: the MBI (upper) included personal hygiene and feeding, whereas the MBI (lower) included ambulation and stair climbing. Spearman’s rank correlation test was used for all correlation analyses. Statistical significance was defined as *P*-value ≤ .05.

## 3. Results

### 3.1. Demographic data

We reviewed the charts of 70 patients with unilateral hemiplegia and subacute brain injury, who underwent DTI between May 2018 and March 2020. The participant characteristics are described in Table 1. Among the enrolled patients, 39 were diagnosed with intracerebral hemorrhage (mean age, 60.59 ± 21.7 years), 23 with infarction (mean age, 61.88 ± 24.12 years), 4 with subarachnoid hemorrhage (mean age, 60.18 ± 19.1 years), 2 with hypoxic brain injury (mean age, 51 ± 34.0 years), and 2 with brain tumor (mean age, 62.5 ± 12.5 years). 41 were male (mean age, 60.60 ± 43.6 years) and 29 were female (mean age, 60.21 ± 26.7 years). There were 34 (48.5%) right hemiplegia cases and 36 (51.4%) left hemiplegia cases. Mean 20.59 ± 21.64 days from onset was needed for patients in the MEP study, and 31.31 ± 27.15 days from onset for the DTI.

**Table 1:** Baseline demographic and clinical characteristic of patients.

N = 70	No. (%)	Age (yr)
Sex		
Male	41 (58.6)	60.60 ± 43.6
Female	29 (41.4)	60.21 ± 26.7
Diagnosis		
ICH	39 (54.2)	60.59 ± 21.7
Infarction	23 (31.4)	61.88 ± 24.1
SAH	4 (5.7)	60.18 ± 19.1
Hypoxic brain injury	2 (2.8)	51 ± 34.0
Brain tumor	2 (2.8)	62.5 ± 12.5
Hemiplegic site		
Right	34 (48.5)	60.60 ± 26.4
Left	36 (51.4)	60.36 ± 22.6

Values are presented as mean ± standard deviation.

ICH = intracranial hemorrhage, SAH = subarachnoid haemorrhage.

### 3.2. Comparing affected and unaffected side

The affected and unaffected FN, FA DTI, and MEP amplitude differences were evaluated. In addition, the affected and unaffected patients’ MRC scales and HFT determined at discharge were compared (Table 2). All comparisons were significantly different between the 2 groups. In DTI, the FN of the CST was 235.35 ± 35.00 in the affected side and 494.75 ± 42.34 in the unaffected side, and the FA of the CST was 0.43 ± 0.28 and 0.64 ± 0.09 in the unaffected side. In TMS, the MEP amplitude of FDI was 0.42 ± 0.20 in the affected side and 1.01 ± 0.50 in the unaffected side, and the MEP amplitude of TA was 0.24 ± 0.20 and 0.56 ± 0.37. The MRC of FDI was 2.13 ± 1.53 in the affected side and 4.19 ± 1.01 in the unaffected side, and the MRC of TA was 2.14 ± 1.46 and 3.63 ± 1.58. The grip power was 7.05 ± 5.33 in the affected side and 21.06 ± 13.75 in the unaffected side, the B&B was 9.59 ± 3.71 and 27.36 ± 16.97, and the NHPT was 81.21 ± 75.15 and 51.00 ± 33.32.

**Table 2:** Comparing affected and unaffected sides.

	Affected	Unaffected	*P*-value
FN of the CST	235.35 ± 35.00	494.75 ± 42.34	<.001
FA of the CST	0.43 ± 0.28	0.64 ± 0.09	<.001
MEP amplitude of FDI	0.42 ± 0.20	1.01 ± 0.50	<.001
MEP amplitude of TA	0.24 ± 0.20	0.56 ± 0.37	<.001
MRC of FDI	2.13 ± 1.53	4.19 ± 1.01	<.001
MRC of TA	2.14 ± 1.46	3.63 ± 1.58	<.001
Grip power	7.05 ± 5.33	21.06 ± 13.75	<.001
B&B	9.59 ± 3.71	27.36 ± 16.97	<.001
NHPT	81.21 ± 75.15	51.00 ± 33.32	.004

Values are presented as mean ± standard deviation.

### 3.3. Optimal cutoff value of the FA and FN ratio of the CST

**Figure 1.**
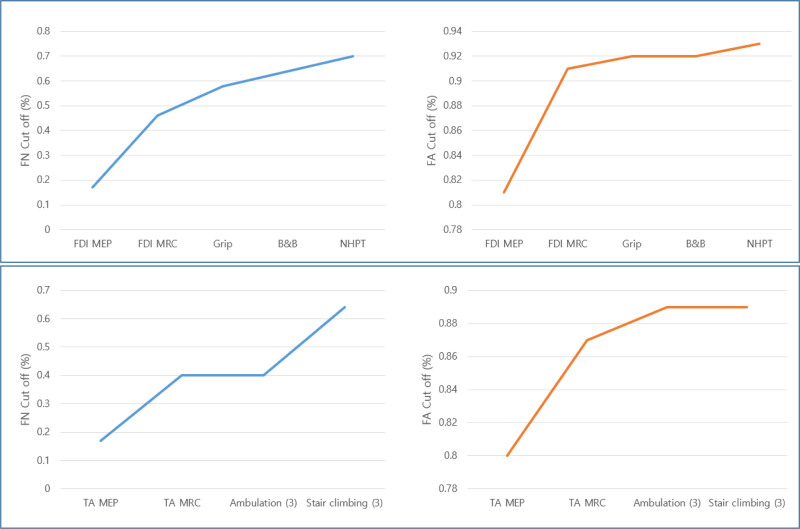
Cutoff values of the corticospinal tract (CST) fiber number (FN) and fractional anisotropy (FA) ratios for upper- and lower-limb motor outcomes. A) cutoff values of CST integrity indices (FN and FA ratios) derived from ROC analysis for upper-limb functions. Higher FN and FA ratios corresponded to better motor performance, including evoked FDI MEP, MRC grade ≥ 2 of the FDI, measurable grip strength, Box and Block Test (B&B), and Nine-Hole Peg Test (NHPT). (B) cutoff values of CST integrity indices (FN and FA ratios) for lower-limb functions. Higher FN and FA ratios were associated with better motor performance, including evoked TA MEP, MRC grade ≥ 2 of the tibialis anterior (TA), and higher ambulation and stair-climbing grades on the Modified Barthel Index (MBI). Abbreviations: FDI, first dorsal interosseous; MEP, motor-evoked potential; FN, fiber number; FA, fractional anisotropy; CST, corticospinal tract; MRC, Medical Research Council; B&B, Box and Block Test; NHPT, Nine-Hole Peg Test; TA, tibialis anterior; MBI, Modified Barthel Index; Ambulation (3) and Stair climbing (3), MBI grade ≥ 3 for each item (corresponding to an item score, indicating moderate assistance or better).

For the upper extremity, the optimal cutoff values of the FN ratio were 0.17 for patients with evoked FDI MEPs, 0.46 for those with finger flexion MRC ≥ 2, 0.58 for those with measurable grip strength, 0.64 for those who could perform the B&B test, and 0.70 for those who could perform the NHPT. The optimal cutoff values of the FA ratio were 0.81, 0.91, 0.92, 0.92, and 0.93 for the respective functions (Table 3, Fig. 1A).

**Table 3:** Optimal cutoff scores for the FDI MEP amplitude ratio, FN ratio, and FA ratio, when the hand function tests could be determined.

FN ratio	Cutoff	Sensitivity (%)	Specificity (%)	AUC
Evoked FDI MEP	0.17	90.5	71.4	0.857
MRC of FDI ≥ 2	0.46	82.1	76.9	0.854
Measurable Grip	0.58	73.1	80.0	0.823
Performable B&B	0.64	73.7	74.5	0.789
Performable NHPT	0.70	80.0	76.5	0.830

FA ratio	cutoff	Sensitivity (%)	Specificity (%)	AUC
Evoked FDI MEP	0.81	88.1	81.5	0.942
MRC of FDI ≥ 2	0.91	82.1	76.9	0.859
Measurable Grip	0.92	80.8	80.0	0.905
Performable B&B	0.92	73.7	68.1	0.797
Performable NHPT	0.93	73.3	68.6	0.795

AUC = area under the curve, B&B = Box and Block test, FA = fractional anisotropy, FDI = first dorsal interosseous, FN = fiber number, MRC = Medical Research Council, NHPT = nine-hole peg test.

For the lower extremity, the optimal cutoff values of the FN ratio were 0.17 for patients with evoked TA MEPs, 0.40 for those with ankle dorsiflexion MRC ≥ 2, 0.64 for those who achieved ambulation or stair climbing with moderate assistance or better. The corresponding FA ratio cutoff values were 0.80, 0.87, and 0.89, respectively (Table 4, Fig. 1B). Higher FN and FA ratios of the CST were associated with better motor and functional performance.

**Table 4:** Optimal cutoff scores for the TA MEP amplitude ratio, FN ratio, and FA ratio when MBI sub-score could be determined.

FN ratio	Cutoff (%)	Sensitivity (%)	Specificity (%)	AUC
Evoked TA MEP	0.17	88.4	70.4	0.880
MRC of TA ≥ 2	0.40	71.9	78.6	0.767
Ambulation (3)	0.40	81.3	77.1	0.811
Stair climbing (3)	0.64	68.8	68.6	0.771

FA ratio	cutoff (%)	Sensitivity (%)	Specificity (%)	AUC
Evoked TA MEP	0.80	83.7	73.1	0.856
MRC of TA ≥ 2	0.87	71.9	72.9	0.738
Ambulation (3)	0.89	71.9	71.4	0.744
Stair climbing (3)	0.89	75.0	60.8	0.667

AUC = area under the curve, FA = fractional anisotropy, FN = fiber number, MRC = Medical Research Council, TA = tibialis anterior.

### 3.4. Correlation between CST metrics, MEP, and functional scales

**Figure 2.**
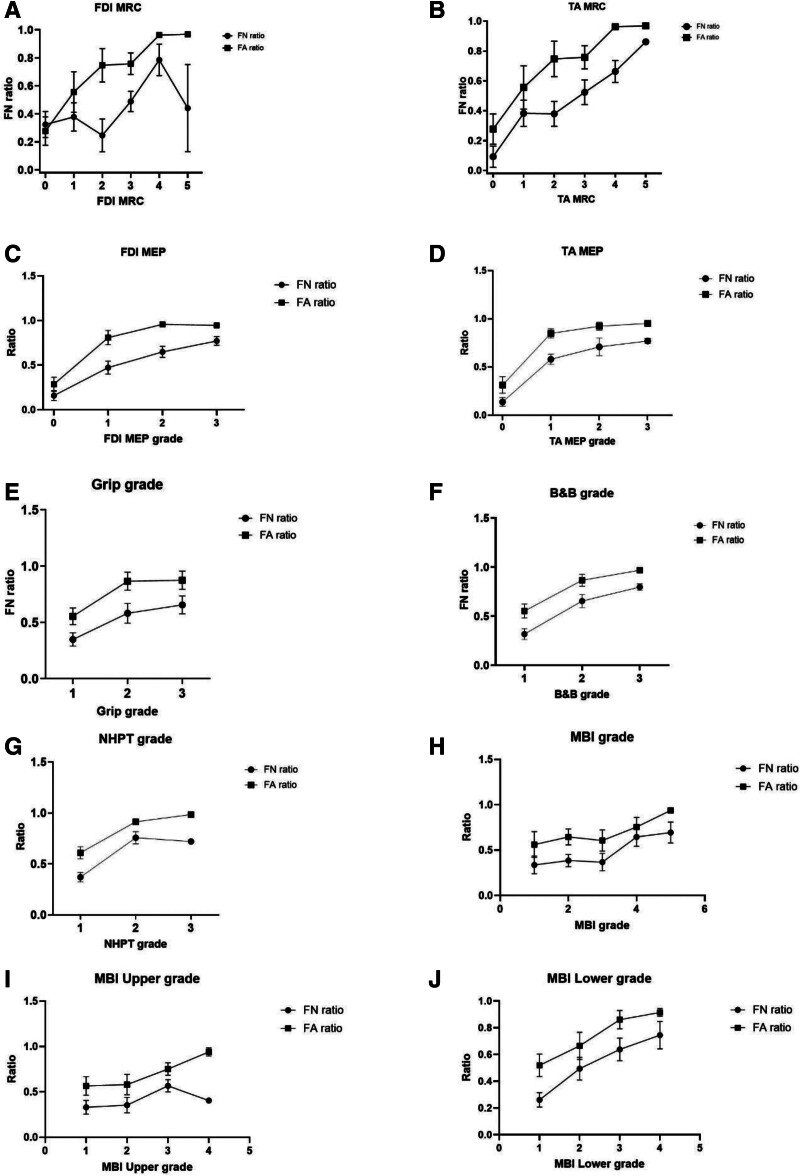
Association of Corticospinal Tract Metrics(FA and FN Ratios) With Categorized Motor Function Outcomes. (A–B) FN and FA ratios according to FDI MRC and TA MRC grades (Medical Research Council scale 0–5). (C–D) FN and FA ratios across FDI MEP and TA MEP grades, categorized into 4 amplitude-based levels: no response (0 mV), low (0–0.5 mV), moderate (0.5–1.0 mV), and good (>1.0 mV). (E–G) Upper-limb function assessed by Grip strength, Box and Block (B&B), and Nine-Hole Peg Test (NHPT), categorized into 3 performance levels: weak/poor/slow, moderate/fair, and good/normal according to established clinical criteria. (H–J) Functional independence evaluated by Modified Barthel Index (MBI), including total MBI, MBI Upper, and MBI Lower grades. MBI total scores were divided into 5 levels: total dependence (0–24), severe (25–49), moderate (50–74), slight (75–90), and independent (91–100). Upper- and lower-limb subscores were each classified into 4 stages: severe (0–4), moderate (5–9), slight (10–14), and independent (≥15). Data are expressed as mean ± SEM. Both FA and FN ratios showed progressive increases with higher functional grades, indicating that greater CST integrity was associated with better motor and functional recovery. CST, corticospinal tract; FA, fractional anisotropy; FN, fiber number; FDI, first dorsal interosseous; TA, tibialis anterior; MRC, Medical Research Council; MEP, motor-evoked potential; B&B, Box and Block Test; NHPT, Nine-Hole Peg Test; MBI, Modified Barthel Index; SEM, standard error of the mean.

Both FN and FA ratios of the CST were significantly correlated with MEP amplitudes of the FDI and TA, MRC grades, HFT results, and MBI scores (Fig. 2, Table 5).

**Table 5:** Correlation between FN & FA of CST, MEP, and functional scales.

FN ratio	MEP Amp. ratio of FDI	MEP Amp. ratio of TA	MRC of FDI	MRC of TA	Grip power	B&B	NHPT	MBI (Upper)	MBI (Lower)
	*R* = 0.551	*R* = 0.410	*R* = 0.428	*R* = 0.369	*R* = 0.269	*R* = 0.348	*R* = 0.148	*R* = 0.332	*R* = 0.368
	*P* < .001	*P* = .001	*P* < .001	*P* = .002	*P* = .040	*P* = .005	*P* = .262	*P* = .006	*P* = .002

FA ratio	MEP Amp. ratio of FDI	MEP Amp. ratio of TA	MRC of FDI	MRC of TA	Grip power	B&B	NHPT	MBI (Upper)	MBI (Lower)
	*R* = 0.529	*R* = 0.475	*R* = 0.524	*R* = 0.464	*R* = 0.334	*R* = 0.352	*R* = 0.209	*R* = 0.269	*R* = 0.359
	*P* < .001	*P* < .001	*P* < .001	*P* < .001	*P* = .010	*P* = .005	*P* = .111	*P* = .003	*P* = .035

B&B = box and block, FA = fractional anisotropy, FDI = first dorsal interosseous, FN = fiber number, MBI = Modified Barthel Index, MEP = motor-evoked potential, MRC = Medical Research Council, NHPT = Nine-hole peg test, TA = tibialis anterior.

## 4. Discussion

This study showed that the FA ratio of the CST may be an objective parameter to assess functional motor levels in patients with subacute brain injury. The MEP amplitude and functional assessment differed between the affected and unaffected sides. The FN and FA of the CST also differed between the affected and non-affected sides. The optimal cutoff values for the FN and FA ratios of the CST were obtained based on whether the MEP was evoked and whether the functional scale was performed; higher FN and FA ratios of the CST were observed in cases of more difficult fine motor function and more difficult ambulation. The FN and FA ratios of the CST correlated with MEP amplitude and functional assessment.

Recently, DTI has been found useful to evaluate the CST in patients with brain injury. Tractography, which assesses the integrity of neural fibers, has the advantage of CST visualization. It can be used to predict motor recovery after both ischemic stroke and intracerebral hemorrhage, and the DTT values reflect the degree of CST.^[17–20]^

The FN of the DTT metric represents the number of voxels included in the neural tract.^[21]^ Therefore, a lower FN of the DTT metrics represents a more severe impairment of the neural and CSTs. In previous studies, the FN of neural tracts showed a positive correlation with motor function in stroke patients.^[12,25,26]^ In our study, the FN of CST ratio cutoff values among patients who showed positive MEP responses was the lowest, and the FN of CST ratio cutoff values among patients who could perform all of the HFTs and the K-MBI ambulation test was higher for the more sophisticated movements of the HFTs and for more difficult ambulation.

The FA of DTI indicates the degree of directionality of water diffusion, and reduced FA of DTI is associated with impaired integration of the neural tract.^[27,28]^ In a previous study, FA values were used to predict motor outcomes in patients with intracerebral hemorrhage,^[29]^ and another study predicted motor function using the FA ratio.^[30,31]^ Similar to the FN of the CST ratio, the FA of the CST ratio cutoff values among patients who showed positive MEP responses was the lowest, and the FA of the CST ratio cutoff values among patients who could perform all of the HFTs and the K-MBI ambulation test was higher for the more sophisticated movements of the HFTs and for more difficult ambulation. This can be combined with the hypothesis that patients with a higher FN and FA ratio of the CST can perform more challenging functional activities with the upper and lower extremities, and that the FN and FA ratio of the CST can be an objective indicator to assess motor function.

Damage to the motor homunculus, which represents a somatotopic organization of the body within the primary motor cortex, plays a crucial role in motor function. The motor homunculus is organized such that the lower limb is represented medially along the paracentral lobule, whereas the upper limb and face are represented laterally along the precentral gyrus.^[32]^ Lesions involving the lateral “hand knob” region of the precentral gyrus have been shown to cause contralateral fine motor deficits such as impaired finger dexterity and grip strength,^[33]^ while medial cortical lesions involving the paracentral lobule primarily result in weakness of the lower extremities and gait disturbance.^[34]^ Given this somatotopic organization, the integrity of CST fibers originating from specific regions of the motor homunculus corresponds to distinct motor functions. Therefore, in this study, we analyzed the FN and FA of the CST by dividing the ROIs into those corresponding to the hand knob and lower limb motor cortex. However, no statistically significant difference was observed according to lesion location. Because the ROIs we delineated included the entire motor homunculus, it was difficult to isolate specific upper or lower limb representations within the CST. Nonetheless, our findings suggest that higher FN and FA ratios of the CST reflect preserved microstructural integrity that enables more complex fine motor control rather than only simple gross movement.

The difference between our study and previous DTI studies is that we used the ratio of the DTI metrics. DTI is particularly sensitive to artifacts generated by eddy currents, motion, and susceptibility, which cause ghosting and geometric distortions.^[35]^ Tractography renders it possible to use personal techniques, and the frequent use of tractography has led to improvements in the reconstruction method.^[36]^ Due to these limitations, we hypothesized that the absolute values of the DTI metrics may be different for each individual. Therefore, we used the ratio of DTI metrics based on the unaffected side. Although there have been few studies using the ratio of DTI metrics,^[37]^ in our study, we used not only the correlation between the ratio of the DTI metrics and functional assessment, but also the correlation with MEP, and we used ROC curves according to functional assessment difficulty.

Since the absolute value of the FA of CST of the affected hemisphere and the unaffected hemisphere did not demonstrate much difference compared to the FN of CST, the difference of FA of the CST ratio cutoff value was subtle among patients who showed finger flexion and ankle dorsiflexion MRC scale ≥ grade 2 and who could perform HFT and ambulation. A previous study reported that the FN of the CST was higher in patients who were able to walk independently than in patients who could not walk and in normal control subjects without changes in the FA of the CST.^[25]^ From this perspective, FN could serve as a complementary parameter that provides additional information when evaluating detailed aspects of motor function, particularly in cases where FA changes are subtle.

The FN and FA ratios of DTI significantly correlated with the MEP amplitude ratio and functional assessment. The FN and FA ratios of DTI refer to the relative differences in the number of voxels and integration of the neural tract in the bilateral hemispheres. Therefore, compared to the undamaged side, it could be considered a ratio that could demonstrate the degree of damage to the damaged cortical spinal tract. Considering the close relationship between functional assessment and DTI values, the FN and FA ratios of the CST seem to represent the patient’s functional movement of dexterity. Additionally, we found that higher FN and FA CST ratios were associated with the possibility of conducting more sophisticated hand movements and more difficult ambulation. However, the correlation pattern was not entirely linear across all functional categories. In particular, the FN ratio tended to decrease in the FDI MRC grade 2 and grade 5 groups, and a similar decline was observed in the MBI Upper group 4 and NHPT group 3, despite the overall positive trend. These fluctuations are likely attributable to the small number of subjects in these specific subgroups, which reduced statistical robustness and representativeness.

Of the 70 enrolled patients in this study, 4 patients did not have a visible CST extending to the motor cortex on the affected side, yet their finger flexion MRC scale score was grade 3. Of these 4 patients, 2 were evoked in the TMS study and 2 were not evoked. Many studies have attempted to understand the correlation between TMS and DTT and motor outcomes after stroke.^[25,38]^ In one study, TMS showed higher positive predictability and DTT showed higher negative predictability in terms of predicting motor outcomes in patients with infarction.^[38]^ Our study also showed that TMS had higher positive predictability than DTT. Although our study focused on the FN and FA of the CST, it is thought to be advantageous to predict motor outcomes using DTT and TMS simultaneously.

This study has several limitations. First, diffusion tensor imaging (DTI) and TMS assessments were performed only once during the subacute stage, and the timing of these evaluations varied among patients. This variability may have influenced the results, as both motor recovery and CST integrity can change dynamically during this phase. Nevertheless, all assessments were conducted within the subacute period (7–90 days after onset), which is considered a critical window for neuroplasticity and functional recovery. No follow-up imaging was performed to monitor longitudinal changes in CST integrity. Second, our study population was heterogeneous, including patients with various etiologies such as ischemic stroke, intracerebral hemorrhage, subarachnoid hemorrhage, hypoxic brain injury, and brain tumors. While large MCA strokes often disrupt the primary motor cortex and descending CSTs, conditions such as traumatic brain injury, hypoxic brain injury, or brain tumors tend to show variable degrees of CST involvement depending on lesion location and severity.^[4–7,10,39]^ Although this heterogeneity could reduce the uniformity of pathophysiological mechanisms, it was intentionally incorporated to enable a broader comparative analysis of motor function according to CST integrity across diverse forms of acquired brain injury. Moreover, most stroke cases in our cohort involved the MCA territory, where CST involvement is particularly common, whereas non-stroke cases generally exhibited less direct invasion of the primary motor cortex. This inclusive design reflects real-world neurorehabilitation settings and strengthens the generalizability of our findings on CST-related motor impairment across different etiologies. Third, the data were collected retrospectively from patients admitted to the Department of Rehabilitation Medicine at Jeonbuk National University Hospital (Jeonju, South Korea) between January 2020 and December 2022, where the patient population was primarily of Asian ethnicity. These factors may limit the generalizability of the findings to other institutions or regions. Fourth, because of the retrospective nature of this study, there may have been uncontrolled confounding factors, such as variability in rehabilitation intensity, comorbid conditions, which could have influenced the results. Therefore, future prospective, multicenter clinical studies with larger sample sizes and serial follow-up assessments are warranted to validate whether the FN and FA ratios of the CST can serve as reliable predictors of motor recovery and functional prognosis across different stages of brain injury.

## 5. Conclusion

This study aimed to investigate whether DTT data are sufficiently objective to assess motor function in patients with subacute brain injury. The FN and FA of the CST significantly correlated with MEP amplitude and functional assessment. Higher FN and FA CST ratios indicate the possibility of performing more sophisticated hand movements and more difficult ambulation activities, thereby suggesting that FN and FA CST ratios may be useful in predicting motor function in patients with subacute brain injury.

## Acknowledgments

The authors extend their appreciation to all members of the Department of Physical Medicine & Rehabilitation at Jeonbuk National University Hospital.

## Author contributions

**Conceptualization:** Myoung-Hwan Ko, Gi-Wook Kim.

**Data curation:** Choong-Hee Roh, Yu Hui Won, Sung-Hee Park.

**Formal analysis:** Choong-Hee Roh, Yu Hui Won, Gi-Wook Kim.

**Funding acquisition:** Myoung-Hwan Ko.

**Investigation:** Myoung-Hwan Ko, Da-Sol Kim, Jeong-Hwan Seo, Gi-Wook Kim.

**Methodology:** Myoung-Hwan Ko, Gi-Wook Kim.

**Project administration:** Gi-Wook Kim.

**Resources:** Myoung-Hwan Ko, Da-Sol Kim, Yu Hui Won, Sung-Hee Park, Jeong-Hwan Seo, Gi-Wook Kim.

**Software:** Myoung-Hwan Ko, Sung-Hee Park, Gi-Wook Kim.

**Supervision:** Gi-Wook Kim.

**Validation:** Choong-Hee Roh, Myoung-Hwan Ko, Gi-Wook Kim.

**Visualization:** Choong-Hee Roh.

**Writing – original draft:** Au-Jin Wang, Choong-Hee Roh.

**Writing – review & editing:** Au-Jin Wang, Gi-Wook Kim.
